# Blood Focused-Metabolomics and Transcriptomics Uncover Non-Linear Risk Association of Inadequate Dietary Choline Intake-Linked Metabolic Stress with MASLD Through Amino Acid Biomarkers, *BCAA* and *MTORC 1*/*AKT1*/*IRS1* Mechanistic Mediators: A Nested Case–Control Study

**DOI:** 10.3390/ijms27104186

**Published:** 2026-05-08

**Authors:** Chien-Hsien Wu, Ming-Lu Lin, Chao-Yun Wang, Chi-Yang Chang, Fu-Jen Lee, Mei-Ling Cheng, Yu-Shun Lin, Tong-Wei Chen, Yi-Ting Hsiao, Bei-Wen Wang, Chang-Sheng Kuo, Rwei-Fen S. Huang

**Affiliations:** 1 Ph.D. Program in Nutrition and Food Science, Fu Jen Catholic University, New Taipei City 242062, Taiwan; jasper5j@yahoo.com.tw (C.-H.W.); fjuns371@gmail.com (C.-Y.W.);; 2 Department of Gastroenterology and Hepatology, Taipei Hospital, Ministry of Health and Welfare, New Taipei City 242033, Taiwan; 3 Department of Nutritional Science, Fu Jen Catholic University, New Taipei City 242062, Taiwan; mll0312@mail.dyu.edu.tw (M.-L.L.); yslin@mail.cmu.edu.tw (Y.-S.L.);; 4 Department of Biomedical Engineering, Dayeh University, Changhua City 51591, Taiwan; 5 Division of Gastroenterology and Hepatology, Department of Internal Medicine, Fu Jen Catholic University Hospital, New Taipei City 242062, Taiwan; 138592@mail.fju.edu.tw (C.-Y.C.);; 6 Metabolomics Core Laboratory, Healthy Aging Research Center, Chang Gung University, Taoyuan 33302, Taiwan; chengm@gap.cgu.edu.tw; 7 Department of Nutrition, China Medical University, Taichung City 406040, Taiwan; 8 Department of Nutrition, Fu Jen Catholic University Hospital, New Taipei City 243089, Taiwan

**Keywords:** choline metabolic stress, metabolic dysfunction-associated steatosis liver disease, plasma-free amino acid, BCAA catabolism, mTOR/Akt/IRS1 signaling

## Abstract

Inadequate choline intake-induced choline metabolic stress (CMS) has been divergently linked to metabolic dysfunction-associated steatotic liver disease (MASLD), yet underlying mechanisms remain unclear. We hypothesized that CMS modifies plasma-free amino acid (PFAA) signatures to influence MASLD risk. In a nested case–control study of 125 participants, dietary choline intake and blood choline metabolites were assessed together with targeted metabolomics and transcriptomic profiling. MASLD was characterized by low choline intake, reduced plasma betaine/choline ratio (Pbcr), elevated homocysteine, increased branched-chain amino acids (BCAAs), and depleted serine/glycine, achieving strong predictive accuracy (AUC = 0.90). CMS was associated with reduced lymphocytic transcripts involved in BCAA catabolism and altered mTORC1/Akt/IRS1 signaling. Nonlinear Pbcr- and intake-dependent MASLD risk patterns were attenuated after adjustment for genetic–metabolite networks. These findings identify CMS-responsive metabolic mediators supporting precision choline interventions.

## 1. Introduction

Metabolic dysfunction-associated steatotic liver disease (MASLD) is among the leading causes of many chronic liver diseases, including steatosis, steatohepatitis, cirrhosis, and hepatocellular carcinoma [1,2]. MASLD is a major contributor to global morbidity and mortality, and its prevalence is closely connected to the rising incidences of obesity, type 2 diabetes, and metabolic syndrome [3]. The pathogenesis of MASLD is likely multifactorial and has known associations with abnormal adiposity [4,5,6,7], insulin resistance [8], hyperlipidemia [9], systematic inflammation, and gut dysbiome [10,11]. Given the complex connection of MASLD to multiple metabolic disorders, no effective pharmacotherapy to improve prognosis and support prevention has yet been approved [3]. Current treatments for MASLD typically involve body fat reduction through caloric restriction and physical activity [12]. Precision nutrition strategies to offset MASLD-related metabolic disorders and minimize MASLD risk are thus urgently needed.

Choline is an essential nutrient that serves as a precursor and signaling molecule in multiple biochemical pathways to regulate hepatic lipid homeostasis, energy metabolism, and adiposity [13]. In the liver, choline is a lipotropic nutrient, and choline deficiency can impair DNA methylation, lipogenesis, and the synthesis of membrane phospholipids and very-low-density lipoprotein for excess hepatic lipid exportation [14,15]. Preclinical studies in rodents have documented the causal effect of choline deficiency on abnormal hepatic fat accumulation-associated pathological progression of MASLD [16,17,18].

Human choline requirements are highly individualized and are involved in multifaceted factors such as sex, body mass index (BMI), and the presence of polymorphisms in key genes of choline/folate metabolism [10,19]. Choline metabolic stress (CMS), caused by inadequate choline intake, decreases choline bioavailability through dysbiosis, producing unfavorable blood metabolites of trimethyl N-oxide (TMAO) and homocysteine (Hcy) [20,21]; and cardiometabolic factors, such as obesity and insulin resistance, have been associated with liver damage in humans [22,23]. Studies involving comprehensive estimates of CMS in individuals with MASLD remain limited, and univariate estimates of CMS-related MASLD risk are controversial. For example, an inverse association between MASLD and dietary choline intake was observed among normal-weight women in a cross-sectional study of middle-aged and older Chinese adults, dependent on sex and adiposity [24]. In a hospital-based case–control study, low choline intake predicted increased odds ratios (ORs) of visceral-obesity-related hepatosteatosis [25]. No association between choline intake and the degree of hepatic steatosis was observed in a large cohort of patients with MASLD, whereas in a cohort of postmenopausal women, those with <50% of the adequate choline intake had more substantial fibrosis [26]. A 2017–2020 study by the National Health and Nutrition Examination Survey revealed no overall link between total choline intake and MASLD prevalence [27], whereas higher choline intake within an MASLD group was unexpectedly linked to worse liver fibrosis in men and individuals with overweight or moderate obesity [27]. Choline and betaine concentrations in plasma have been proposed as robust markers that predict dietary choline intake in healthy individuals [28,29]. High plasma choline (Pch) levels in patients with metabolic disorders have been associated with more severe fatty liver and fibrosis [30]. Among middle-aged and older individuals, high blood choline and low plasma betaine levels (Pbet) have been associated with an unfavorable cardiometabolic risk profile of elevated blood triglycerides, glucose, BMI, and obesity—the key components of MASLD [31]. These mixed results regarding the divergent association of CMS with MASLD risk suggest a “double-edged sword” effect of choline nutrition in relation to multifactorial MASLD pathogenesis. The mechanistic mediators contributing to divergent CMS-associated MASLD risk thus remain poorly understood.

Technological advances in blood-focused metabolomics have revealed changes in plasma-free amino acids (PFAAs) profiles that serve as surrogate biomarkers in patients with early-stage and progressive MASLD and various MASLD subtypes. In a discovery cohort of 2000 healthy Japanese participants and a validation cohort of 2160 participants, Yamakado et al. [32] identified an altered PFAA profile associated with fatty liver disease and comorbid metabolic risk factors with high diagnostic accuracy. In particular, the concentrations of circulating branched-chain amino acids (BCAAs), such as leucine, isoleucine, and valine, and of glutamate and tyrosine are often high in patients with MASLD/metabolic dysfunction-associated steatohepatitis (MASH) [32,33]. Elevated blood BCAAs are a well-documented plasma indicator of abdominal obesity, insulin resistance, and hepatic fat accumulation [34,35,36,37,38,39,40,41]. Conversely, patients with these conditions frequently exhibit low concentrations of amino acids, such as glycine and serine. Altered blood PFAA profiles have been associated with obesity [42], diabetes [43], and insulin resistance-related fatty liver [44]. The changes in intracellular and extracellular PFAA concentrations observed in patients with MASLD largely reflect the consequences of multiple organism-driven metabolic dysfunction [34,35,36,37,38,39,40,41]. Given the fact that hepatic choline–betaine–folate cycle involves serine/glycine/methionine metabolism, it raises the possibility that CMS may interact with PFAA metabolism, serving as a surrogate marker of MASLD, to differentially modify the association of fatty liver risk; however, such an interaction has not yet been comprehensively investigated.

To test the hypothesized interaction, we conducted a nested case–control study to establish a CMS threshold, identify CMS-sensitive and MASLD-responsive PFAA markers, and explore related mediators in MASLD risk prediction. The identification of CMS-responsive and MASLD-sensitive PFAA signatures and their interactive mediators can provide mechanistic insights to develop a precision nutrition basis for early detection of disease-related inefficient metabolic patterns in human intervention trials before CMS-induced organ dysfunction and fatty liver become apparent.

## 2. Results

### 2.1. Targeted Metabolomic Profiling of MASLD-Sensitive PFAAs in Study Participants

**Figure 1:**
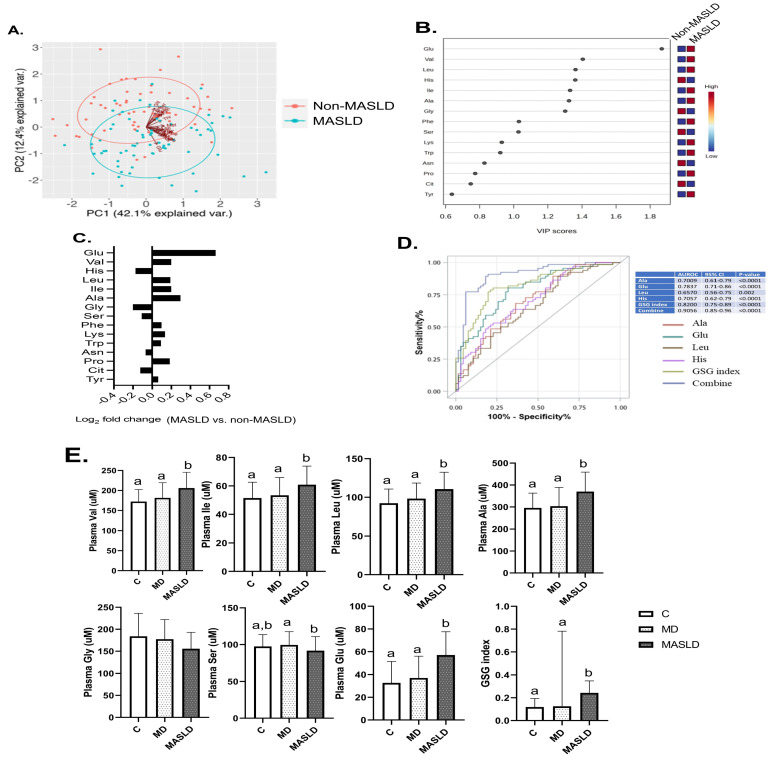
**Targeted metabolomic profiling of MASLD-sensitive PFAAs in study participants.** Discrete PFAA signatures in patients with and without MASLD were analyzed through (**A**) principal component analysis; (**B**) orthogonal partial least squares–discriminant analysis and VIP scoring; (**C**) fold-change analysis (selection criteria: VIP score > 1, *p* < 0.05, R < 0.05, and fold-change > 2); and (**D**) ROC curve of selected PFAAs for MASLD prediction plotted with GraphPad Prism 9. (**E**) MASLD-sensitive PFAA signatures in healthy control participants, patients with metabolic disorders, and patients with MASLD. PFAA data were log-transformed, and differences between subgroups were analyzed through one-way analysis of variance with Tukey’s post hoc test. Values with different letters differ, with significance at *p* < 0.05.

We performed targeted metabolomic analysis of participants’ PFAA profiles. The principal component analysis plot in Figure 1 displays a clear separation of PFAAs between patients with and without MASLD (Figure 1A). Through orthogonal partial least squares–discriminant analysis (OPLS-DA) and variable importance in projection (VIP) scores, we identified glutamate (Glu) as the variable that most distinguished patients with MASLD from those without MASLD, followed by BCAAs, glycine (Gly), serine (Ser), histidine (His), and phenylalanine (Phe) (Figure 1B). Fold-change analysis from the volcano plot (Supplementary Figure S1) demonstrated that patients with MASLD had significantly elevated blood Glu, Ala, and BCAAs and decreased blood Ser, Gly, and His relative to patients without MASLD (Figure 1C). Multiple ROC curve analysis was conducted for 25 PFAAs identified in participant samples to identify significant predictors of MASLD (Figure 1D). Among PFAAs, the AUC for Ala, Glu, Leu, and His was 0.70, 0.78, 0.65, and 0.70, respectively. The GSG index (Glu/Ser + Gly) yielded an AUC of 0.82, indicating good discriminatory capacity. When these four PFAAs were combined with the GSG index, the AUC increased to 0.9 (95% CI: 0.85–0.96, *p* < 0.0001), indicating excellent predictive accuracy. The composition signatures associated with PFAAs related to MASLD were specific to hepatosteatosis, demonstrating the ability to differentiate patients with MASLD from both control and those with metabolic disorders (Figure 1E).

### 2.2. Identification of Distinctive CMS-Responsive PFAA Signatures in Metabolic Disease and Obesity Subgroups

**Figure 2:**
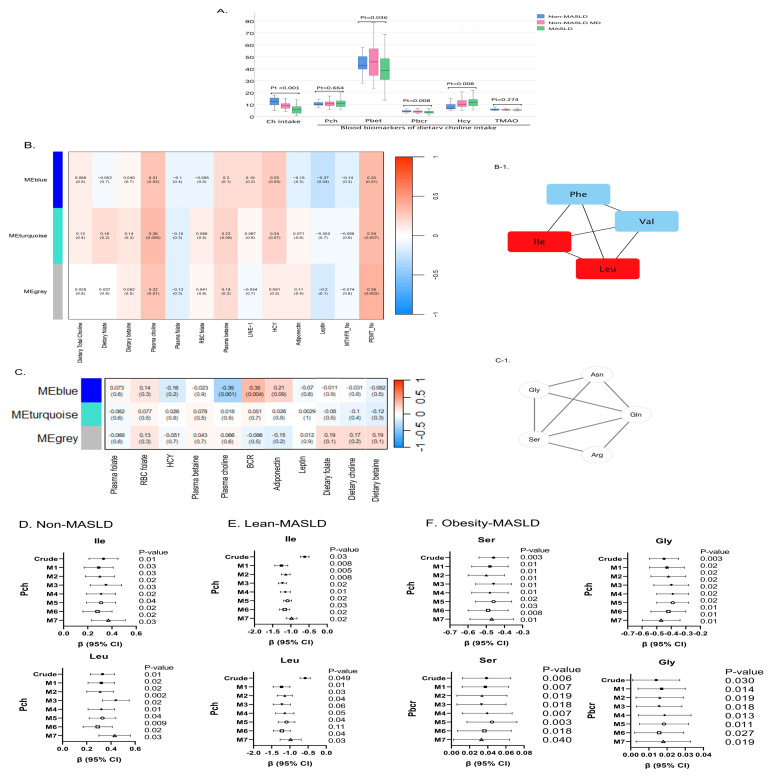
Identification of distinctive CMS-responsive PFAA signatures in the subjects stratified by MASLD and MASLD-associated obesity. (**A**) CMS of participants was characterized using comprehensive dietary and blood choline metabolite markers (Pch, Pbet, Pbcr, Hcy, and TMAO). WGCNA revealed an association of PFAA metabolites with altered CMS in (**B**) non-MASLD and (**C**) MASLD subjects. Each row represents metabolites. Each column represents one-carbon traits and adipokines. Each long square contains a correlation coefficient and *p* value in parenthesis. Differential enriched metabolite networks in non-MASLD and MASLD subjects are indicated in blue (**B-1**,**C-1**) module. Red block indicates hub metabolite. Multivariable linear regression models were constructed to analyze the predictive power of Pch and Pbcr for PFAA signatures in (**D**) non-MASLD subjects, (**E**) lean-MASLD, and (**F**) obesity-MASLD subjects. Obesity criteria are defined as a BMI > 27, a visceral fat level > 10, or a waist-to-hip ratio > 0.9 for men and > 0.85 for women. Multiple adjustment models include age, sex, BMI, dietary energy, and protein intake (Model 1), Hcy (Model 2: M1 + Hcy), insulin resistance (Model 3: M1 + HOMOIR), alanine aminotransferase (ALT; Model 4: M1 + ALT), creatine phosphokinase (CPK; Model 5: M1 + CPK), leptin (Model 6: M1 + leptin), and *PEMT* rs7946 single-nucleotide polymorphism (Model 7: M1 + *PEMT*).

CMS in the study participants was characterized using a comprehensive set of dietary and blood choline metabolite markers (Pch, Pbet, Pbcr, Hcy, and TMAO). Compared with control participants (median: 692 mg/day) and those with metabolic diseases (544 mg/day), participants with MASLD had inadequate choline intake (median: 381 mg/day), which was associated with decreased Pbet and Pbcr and an increased Hcy concentration (Figure 2A). Pch and TMAO levels did not differ among the three groups. Relative to other participants, those with MASLD exhibited a CMS-associated unfavorable cardiometabolic phenotype of elevated ALT, homeostatic model assessment of insulin resistance (HOMOIR), HbA1C, and triglyceride levels and adiponectin/leptin ratio, signifying adipose inflammation (Supplementary Table S3).

We then performed WGCNA to classify the coexpression of CMS traits with PFAA network signatures associated with MASLD. In patients without MASLD, Pch, *PEMT* rs7946 single-nucleotide polymorphism, and blood leptin levels were significantly associated with BCAAs/Phe network signatures, with Leu and Ile as the hub metabolites (Figure 2B,B-1). In patients with MASLD, both Pch (r = −0.39, *p* = 0.001) and Pbcr (r = 0.35, *p* = 0.004) were significantly associated with serine/glycine, arginine/asparagine, and glutamine in MEblue modules (Figure 2C,C-1).

After stratification by obesity criteria, the identified CMS-responsive PFAA signatures were associated with obesity-related MASLD compared with lean or no MASLD [19,22] (Supplementary Table S1). After adjustment for multiple MASLD risk factors, increased Pch significantly predicted elevated BCAA levels (Leu and Ile) in patients without MASLD (Figure 2D). Notably, an inverse association between Pch and Leu/Ile levels was observed in the lean MASLD group (Figure 2E). In patients with obesity and MASLD, elevated Pch predicted depleted plasma levels of serine and glycine (Figure 2F). In patients with lean and obesity-related MASLD, Pbcr was associated with depleted blood Ser/Gly levels (Figure 2F). In patients with obesity-related MASLD, Pbcr was associated with depleted blood Ser/Gly levels (Figure 2F). These findings suggest that altered Pch and Pbcr exert divergent effects on distinctive MASLD-specific PFAA signatures of elevated BCAAs and depleted Ser/GLy levels associated with obesity status.

### 2.3. Targeted Blood Transcriptomic Analysis of Transcriptional Regulators and Signaling Molecules in CMS-Related BCAA Catabolism

**Figure 3:**
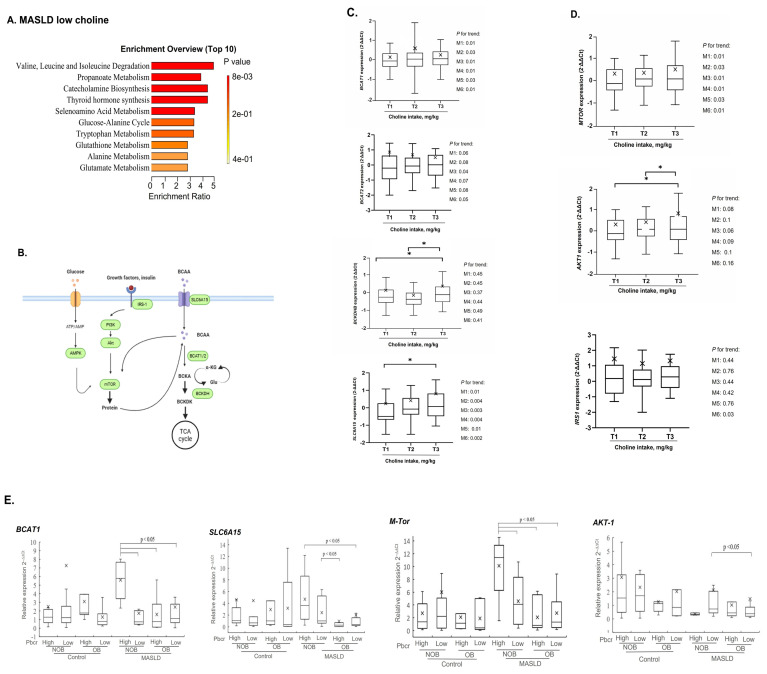
Targeted blood transcriptomic analysis of transcriptional regulators and signaling molecules in CMS-related BCAA catabolism. (**A**) Enrichment pathway analysis of the disease-sensitive PFAA network signatures in patients with MASLD. The metabolites involved in enriched pathways were identified using the Kyoto Encyclopedia of Genes and Genomes (KEGG) database. The MASLD subjects were stratified by high vs. low Pch status with the cutout threshold levels at a medium value (11.5 uM). *p* ≤ 0.05 and enrichment ratio > 1 were used to select the significant enrichment pathway. (**B**) Diagram of key enzymatic regulators and AA-sensing signaling molecules in the BCAA catabolic pathway. Lymphocytic mRNA expression of the key regulators in BCAA catabolism for (**C**) *BCAT1*, *BCAT2*, BCKDHB, and *SLC6A15*), and (**D**) signaling regulators of *mTORC1*, *AKT1*, and *IRS1* axis were stratified by tertiled choline intake (T1: 1.96 ± 0.70 mg/kg BW; T2: 4.70 ± 1.30 mg/kg BW; and T3: 8.30 ± 2.00 mg/kg BW) of patients with MASLD. Multivariable-adjusted models include adjustment for Model 1 (M1: age and sex), Model 2 (M2: M1 + homocysteine (HCY)/trimethylamine N-oxide (TMAO)/betaine-to-choline ratio (BCR), and long interspersed nuclear element-1 (LINE-1)), Model 3 (M3: M1 + aspartate aminotransferase (AST)/alanine aminotransferase (ALT)/AST-to-ALT ratio), Model 4 (M4: M1 + creatine phosphokinase (CPK)), Model 5 (M5: M1 + HOMA-IR), and Model 6 (M6: M1 + blood triglycerides/total cholesterol/adiponectin/leptin, and the adiponectin-to-leptin ratio (A/L ratio)). Blood transcriptional expression of (**E**) *BCAT1*, *SLC6A15*, *mTORC1*, and *AKT1* of the study subjects was stratified by disease stages and obesity status. Transcript levels were analyzed using quantitative real-time polymerase chain reaction. Glyceraldehyde 3-phosphate dehydrogenase was the reference gene. Data are presented as 2^−ΔΔCt^ in participants with versus without MASLD. Differences between subgroups were analyzed through one-way analysis of variance with Tukey’s post hoc test. * *p* < 0.05 indicates statistical significance. *p* for trend: represents the *p* value for linear trend across choline intake tertiles adjusted for body weight.

To obtain biological insight as to the overrepresentation function of the CMS-responsive and MASLD-sensitive metabolite signatures, enrichment analyses were performed (Supplementary Figure S2). As shown in Figure 3A, for the MASLD subjects with low Pch levels, the term “Branched chain amino acids degradation” was the top significantly enriched pathway. Thus, targeted blood transcriptomic analysis was performed to delineate the effects of choline intake-linked CMS on transcript levels of key rate-limiting enzymes in BCAA catabolic enzymes such as branched-chain aminotransferase (*BCAT1* and *BCAT 2*), branched-chain α-keto acid dehydrogenase (*BCKDHB*), and *BCKDH* regulator of branched-chain alpha-keto acid dehydrogenase kinase (*BCKDK*) combined with *SLC6A15* (an amino acid transporter for BCAAs), and signaling regulators in the *mTORC1*/*AMPK*/*AKT1*/*IRS1* axis (Figure 3B).

When choline intake (mg/kg body weight) was tertiled, decreased choline intake was significantly associated with reduced mRNA abundances of *BCAT1* of patients with MASLD- after multiple adjustments for CMS and cardiometabolic risk factors (Ptrend < 0.05) (Figure 3C). As shown in Figure 3C, similar associations of decreased choline intake with reduced transcript abundance of *BCAT2*, and *SLC6A15* were observed in patients with MASLD. T1/T2 vs. T3 intake was significantly associated with reduced mRNA abundance of *BCKDHB*. Adjustment for multiple risk factors negated this association (Figure 3C). Decreased choline intake was associated with reduced abundance of *mTORC1* transcripts (Ptrend < 0.05) and *IRS1* transcript (M6: Ptrend: 0.03) (Figure 3D). Reduced *AKT1* transcripts were associated with decreased choline intake; however, the significance of this association was negated by adjustment for cardiometabolite risk factors (M1–6).

Lastly, we test if Pbcr, a blood marker of altered choline intake, was associated with the blood transcriptional signaling molecules in BCAA catabolism, as with disease stages and obesity status. As shown in Figure 3E, Pbcr was associated with increased mRNA abundance of *BCAT1* among participants with lean MASLD. Relative to participants with lean MASLD, those with obesity and MASLD exhibited reduced *BCAT1* expression.

A similar interaction effect of Pbcr and obesity on transcript expression was observed for *SLC6A15* in patients with MASLD (Figure 3E). Low vs. high Pbcr was associated with reduced mRNA abundances of *mTORC1* (Figure 3E) and increased *AKT1* expression (Figure 3E) in patients with lean MASLD. 

### 2.4. Comprehensive Correlation Heatmaps Identify Distinctive CMS-Responsive Blueprints for OB-Sensitive Blood Metabolites and Transcriptional Coexpression Network Signatures Among Non- and MASLD Subjects

**Figure 4:**
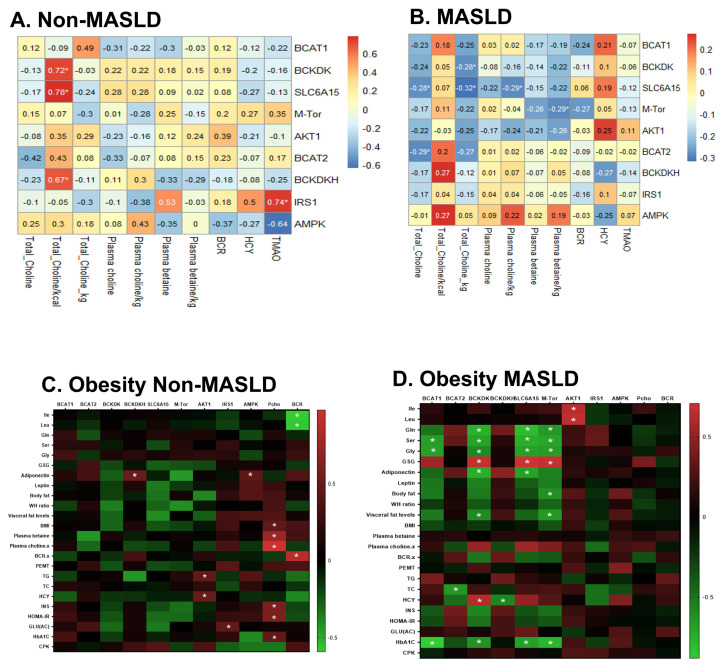
Correlation heatmaps between CMS, blood PFAA and clinical markers, and transcriptional coexpression network signatures stratified by MASLD stage and obesity status. Association of CMS markers with lymphocytic transcriptional signatures among (**A**) non-MASLD and (**B**) MASLD subjects. Functional association of blood transcriptional signatures with CMS, PFAAs, and cardiometabolic markers among (**C**) obesity-non-MASLD and (**D**) obesity-MASLD. * indicates the significant Pearson correlation coefficient at *p* < 0.05. Color scale indicated positive (red) and inverse (blue) correlation.

We constructed a comprehensive correlation heatmap and interactive network to analyze the functional association of blood transcriptional signatures with CMS, PFAAs, and cardiometabolic markers in response to MASLD/obesity status. As shown in Figure 4, the deregulated transcriptional signatures of BCAA catabolism and *mTORC1*/*AKT1*/*IRS1* signaling were significantly correlated with CMS, such as low choline intake, as well as blood metabolites of Pch and Pbet among participants with MASLD (Figure 4B). Among non-MASLD participants, low choline intake and blood TMAO levels were associated with deregulated expression of BCAA catabolism and IRS1 signaling (Figure 4A). Among participants with obesity and MASLD (Figure 4D), the deregulated transcriptional signatures of BCAA catabolism and *mTORC1* signaling were significantly associated with PFAA levels (BCAAs, Glu, Ser, Gly, and GSG index), blood metabolites (Hcy, blood cholesterol, and HbA1C), and body adiposity (body fat and visceral fat). The genetic and PFAA metabolite coexpression network signatures associated with obesity-MASLD were not observed among obese participants without MASLD (Figure 4C). Further heatmap reanalysis showed that such functional correlation between leukocyte transcripts and CMS/blood PFAA/clinical MASLD markers robustly exists in control subjects, metabolic disorder subjects, subjects with low choline intake, and those with high choline intake, using adequate intake (AI) as the cutoff (Supplementary Figure S3).

### 2.5. Risk Threshold and Modifiers of CMS for MASLD Prediction

**Figure 5:**
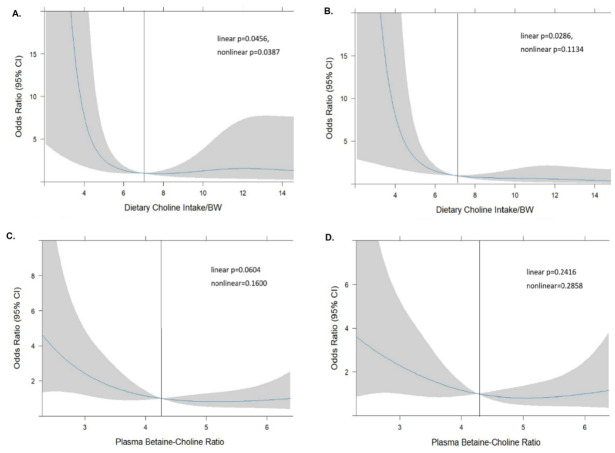
Risk threshold and modifying factors of CMS for MASLD prediction. Restricted cubic spline regression was used to analyze the risk threshold of choline intake (**A**,**B**) and Pbcr (**C**,**D**) for MASLD prediction. The shadow accompanying the regression line represents the 95% confidence interval. Odd ratio for choline intake at the sex-dependent cutout (men: 7.5 mg/day; women: 7.0 mg/day) was adjusted by (**A**) age, energy intake, Hcy, Pbcr, genetic polymorphisms, blood glucose, and HOMOIR, and (**B**) transcriptional regulators of BCAA catabolism. Crude model for the odd ratio of Pbcr and MASLD risk prediction (**C**) was adjusted by age, sex, and obesity (**D**).

We performed restricted cubic spline regression analyses, adjusting for BCAA-related genetic and metabolite coexpression network signatures, to explore the association of CMS with MASLD risk predictors and plausible modifiers. The smooth plot in Figure 5A displays a biphasic curve for choline intake–MASLD risk with significant linear (*p* = 0.045) and nonlinear (*p* = 0.0387) correlations after multiple adjustment. A significant linear trend indicating increased MASLD risk was observed for those with low choline intake (men < 7.5 mg/day; women < 7.0 mg/day). For those with high choline intake above the cutout (>12 mg/kg), increased choline intake was associated with greater risk correlation (nonlinear *p* = 0.038; Figure 5A). Further adjustment for transcriptional regulators of BCAA catabolism strengthened the linear correlation (*p* = 0.028) but abrogated the significant nonlinear correlation (*p* = 0.113) with MASLD risk (Figure 5B). These findings suggest that BCAA metabolism plays a critical role in the biphasic relationship between choline intake and MASLD risk. With Pbcr as the proposed blood marker of dietary choline intake, the smooth plot indicated that Pbcr at a cutoff value of 4.7 predicted an L-shape relationship with MASLD risk. A linear trend of increased MASLD risk was observed for participants with a low Pbcr (linear *p* = 0.06; Figure 5C). Adjustment for age, sex and overweight/obesity negated the linear relationship between Pbcr and MASLD risk among individuals with low Pbcr (Figure 5D). This finding suggests that abnormal adiposity is involved in the association of low blood choline markers with MASLD risk. We also observed an U-shaped curve for Pch–MASLD risk with a significant nonlinear correlation at the cutoff of 10.85 uM after adjustment for age, sex, and BMI (*p* = 0.024). Adjustment for plasma BCAAs and their effect on mTORC1 negated the Pch-based prediction of MASLD risk (Supplement Figure S4). However, huge 95% CI interval observed beyond the Pch cutout made the observed risk relationship unreliable. The negation may be due to statistical artifacts or consequences of unaddressed confounding.

### 2.6. Mechanistic Mediators of the Association Between CMS and MASLD Risk

**Figure 6:**
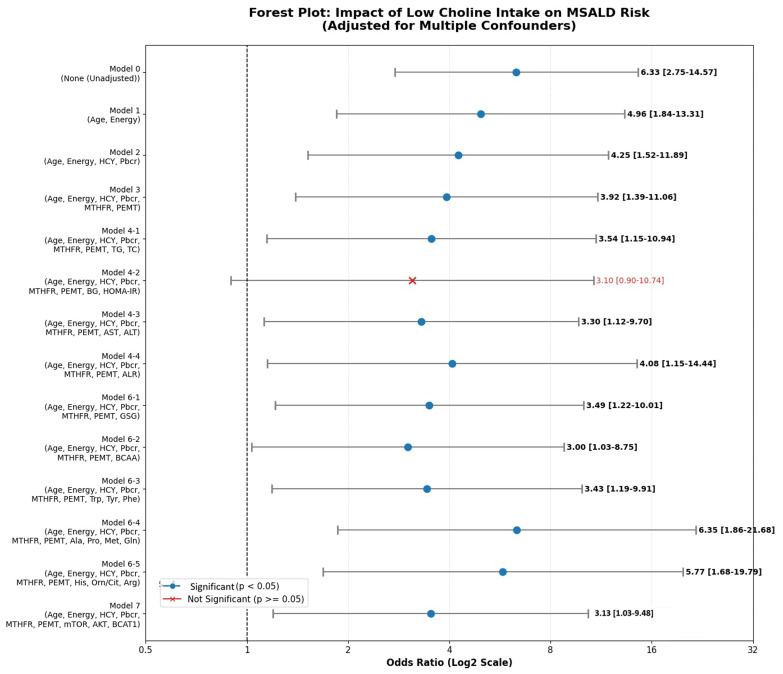
Forest Plot: Impact of Low Choline Intake on MSALD Risk Adjusted for Multiple Confounders.

We constructed binary logistic regression models to explore mediators of the association between low choline intake and MASLD risk. As indicated in the forest plot (Figure 6), low (vs. high) choline intake was associated with a 6.3 increased risk of MASLD (unadjusted crude model). Adjustment for blood markers of choline intake (Hcy + Pbcr; Model 2: OR: 4.25, 95% CI: 1.52–11.8) or polymorphic modifiers of choline biochemical status (*PEMT* + *MTHFR*; Model 3: OR: 3.9, 95% CI: 1.39–11.0) reduced MASLD risk by 39–44%. This finding suggests that disrupted choline metabolism because of insufficient dietary intake and genetic polymorphism of one-carbon enzymes significantly contributes to increased MASLD risk. Adjustment for dyslipidemia (Model 4-1: triglycerides and total cholesterol), liver injury (Model 4-3: ALT and AST), and adipose inflammation markers (Model 4-4: adiponectin/leptin ratio: ALR) did not negate the effect of low choline intake on MASLD risk. Further adjustment for insulin resistance (Model 4-2: blood glucose and HOMOIR) abrogated the significant risk prediction power of low choline intake, indicating that disrupted glucose metabolism is a key mediator of the relationship between choline intake and MALSD risk. Notably, adjustment for blood PFAAs, in particular, Ala, Pro, Gln, and Met (Model 6-4: OR, 6.35; 95% CI, 1.86–21.6) and His, Arg, and Orn/Cit (Model 6-5: OR, 5.77; 95% CI, 1.68–19.7), amplified the effect of low dietary choline on MALSD riskModel adjustment for blood BCAAs (Model 6-2: OR, 3.0; 95% CI, 1.08–8.75) and genetic regulators of BCAA metabolism (*BCAT1*, *BCKDK*, and *SLC6A15*) and bioenergetics–insulin signaling pathways (*mTOR*/*AKT*/*AMPK*/*IRS*; Model 7: OR, 3.13; 95% CI, 1.03–9.48) reduced the effect of low choline intake on MASLDprediction.

This forest plot illustrates the association between low choline intake and the risk of metabolic dysfunction-associated steatotic liver disease (MASLD) across various adjustment models. Axis Scales: The horizontal axis represents the odds ratio (OR) displayed on a Log2 scale. The vertical dashed line indicates an OR of 1.0, representing no observed effect. Data Representation: Blue circles indicate models where the association is statistically significant (*p* < 0.05). Red cross (×) indicates a model (Model 4-2) where the association is not statistically significant. Model Descriptions: Model 0: unadjusted analysis. Models 1–3: successively adjusted for age, energy intake, homocysteine (HCY), Phe, and genetic factors (MTHFR, PEMT). Models 4-1 to 7: further adjusted for metabolic and biochemical variables, including lipids (TG, TC), insulin resistance (HOMA-IR), liver enzymes (AST, ALT), and various amino acid profiles (BCAA, Trp, Tyr, Phe, etc.). Statistical Summary: specific OR values and their corresponding 95% CIs are provided on the right side of the plot for each adjustment level.

### 2.7. Association of Pbcr and Obesity with MASLD Risk Involving Blood Genetics and Metabolite Coexpression Network Signatures

As shown in Table 1, compared with lean individuals with high Pbcr (>4.27), individuals with obesity had a 4.6-fold increased risk of MASLD (Model0: OR, 4.6; 95% CI, 1.1–19.1). The obesity-related MALSD was three times higher in individuals with low Pbcr (Model 1: OR, 15.1; 95% CI, 4.1–54). Adjustment for age, gender (Model 1), and choline intake as well as transcriptional markers (Model 2) did not abrogate the predictive power of Pbcr for obesity-related MASLDrisk. Further adjustment for TG negated the high Pbcr–obesity-related MASLD risk (Model 3), whereas final adjustment for HOMOIR negated the association of low Pbcr–obesity profiles with MASLD risk (Model 4).

## 3. Discussion

Through a comprehensive dietary assessment, we characterized the CMS phenotype of individuals with MASLD. This phenotype is marked by low choline intake, low Pbcr, and high Hcy. Through targeted blood metabolomics, we identified differential CMS involvement in an unfavorable PFAA profile that revealed elevated BCAAs and depleted serine/glycine as MASLD risk predictors, which was dependent on obesity status. Targeted blood transcriptomic profiling further revealed MASLD-specific nutrigenetic features: low choline intake, as indicated by Pbcr values, was associated with reduced transcripts of BCAA-catabolizing enzymes (*BCAT1*, *BCAT2*, *BCKDK*, and *SLC6A15*) and suppressed transcript abundance of *mTORC1*/*AKT*/*IRS* signaling molecules, implying impaired BCAA metabolism. Adjustment for the genetic and metabolic coexpression network signatures of PFAAs negated the relationship between CMS and MASLD, positioning PFAAs as novel, multifaceted variables for precision choline interventions to minimize MASLD risk.

This is the first study to characterize the PFAA-imbalanced phenotype linked to CMS in patients with MASLD as involving depleted Ser/Gly. The identified CMS-responsive phenotypes exhibited alterations in circulating PFAA concentration, characteristic of MASLD/MASH [32,33]. These alterations are well-documented diagnostic biomarkers in patients with early-stage and severe progressive MASLD [25,26,27,28]. In patients with MASLD, low blood glycine or serine often indicates impaired one-carbon metabolism in folate–methionine cycles, which are primarily active in hepatocytes, increasing oxidative stress and inflammation [45]. Diets high in glycine/serine/threonine may slow MASLD progression [33]. In particular, glycine supplementation can reduce oxidative stress and nonalcoholic steatohepatitis in nonhuman primates [46]. Disrupted serine or glycine metabolism often accompanies abnormal glucose homeostasis in selective insulin resistance that contributes to obesity-related MASLD [8,47]. The GSG index is a possible marker of liver disease severity independent of BMI [34]. In a diet-induced MASLD rodent model, dysregulated serine/glycine/methionine metabolism disrupted betaine/choline metabolism, impeding hepatic PC homeostasis, promoting very-low-density lipoprotein formation, and increasing insulin resistance, promoting MASLD [48,49]. Mice with obesity induced by a high-fat diet exhibited abnormal hypermetabolism, which aggravated MASLD when the mice were fed a choline-deficient diet [16,17]. In our patient cohort, the MASLD-specific PFAA signature of depleted glycine or serine was associated with abnormal Pch and Pbcr (CMS markers) and adiponectin (obesity marker). Furthermore, insufficient choline intake was associated with cardiometabolic tissue damage in patients with MASLD, as indicated by elevated ALT (liver damage), HOMOIR (glucose metabolic disorder), HbA1C (oxidative stress), triglycerides (lipid dysfunction), leptin (obesity), and adiponectin/leptin ratio (adipose inflammation), all of which are comprehensive biomarkers of deficient dietary choline intake. After adjustment for cardiometabolic risk factors, including HOMOIR, Pbcr levels independently predicted depleted plasma serine/glycine levels in patients with both lean and obesity-related MASLD. These findings suggest that hepatic choline–betaine cycle metabolism plays a key role in MASLD-related PFAA signatures, dependent on obesity and insulin resistance status. Adjustment for abnormal BMI, blood choline markers (Pbcr and Hcy), insulin resistance, and PFAA markers negated the predictive power of low choline intake for MASLD risk. These novel Pbcr-sensitive, MASLD-responsive PFAA biomarkers may provide mechanistic insight into the inverse relationship between blood betaine, the betaine/choline ratio, and unfavorable obesity-linked risk profiles, which include elevated BMI in older adults [31], patients with acute coronary syndrome [50], young men [51], and in a Newfoundland cohort and a Taiwanese cohort of older adults [52]. Without obesity- and biomarker-based classification, no dietary determinants were associated with circulating choline metabolite levels and disease risk in a pooled population of 32,853 participants from 17 cross-sectional studies [53].

Another novel finding of this study is that elevated blood BCAAs may represent a CMS-responsive, MASLD-sensitive metabolite phenotype. To our knowledge, this finding has not previously been reported in patients with MASLD. Another study involving targeted and nontargeted metabolomic profiling of plasma samples from 53 participants fed baseline, depletion, and repletion choline diets identified decreased metabolite concentration in choline–methionine pathways and in glutamine, glycine, and several BCAAs during choline depletion [54]. Abnormal blood BCAA levels are well-documented indicators of abdominal obesity, hepatic fat accumulation, insulin resistance, and liver disease development [34,35,36,37,38,39,40,41].

The mechanism through which CMS elevates blood BCAA levels remains largely unknown. As such, we performed focused transcriptomic analysis of BCAA metabolism and signaling regulators, revealing several plausible mechanisms. Reduced mRNA abundances in lymphocytic BCAT1 and BCAT2 were significantly associated with low choline intake and a low Pbcr. In another study, loss of BCAT1 and protein BCAT1 in *Bcat1*^*−/−*^ mice affected lipogenesis in the liver, muscle tissue, pancreas, and thymus. The tissue effects of loss of *BCAT2* and BCAT2 resulted in systemic accumulation of blood BCAAs and BCKAs that caused morbidity and mortality in *Bcat2*^*−/−*^ mice [55]. BCAT2 physically interacts with downstream BCKDH to form multienzyme complexes within mitochondria [55], which the inhibitory regulator of BCKDK modulates to control the catabolic flux of BCKA into the TCA cycle for energy metabolism [56,57]. CMS-linked suppression of lymphocytic transcripts of *BCAT1*/*BCAT2* has been correlated with reduced BCKDH and BCKDK expression in patients with MASLD.

The MASLD-specific, CMS-linked elevation of blood BCAAs reduced transcripts of *SLC6A15* in our cohort, which encodes a member of the solute carrier family 6 protein that functions as a sodium-dependent neutral amino acid transporter, with preference for BCAAs and methionine. Impaired activity of this solute carrier suppresses the transport of BCAA substrates across biological membranes, lipid synthesis and metabolism, mitochondrial function, and ferroptosis, all of which contribute to hepatic steatosis and fibrosis development [58]. Elevated blood BCAAs in patients with fatty liver diseases have been attributed to lower BCAA uptake in muscle tissue due to reduced insulin activity associated with insulin resistance and decreased amino acid activity in muscle tissue [59,60]. Additionally, visceral adipose tissues uptake and metabolize blood BCAAs, whereas insulin resistance decreases the expression of BCAA-catabolizing enzymes in adipose tissue. In line with obesity-related BCAA metabolism in adipose tissue [59,61,62], we observed that relative to nonobese patients with MASLD, patients with obesity and MASLD exhibited reduced lymphocytic expression of *BCAT1*, the first rate-limiting enzyme in BCAA catabolism. CMS (low choline intake or Pbcr) was associated with decreased mRNA abundances of lymphocytic *BCAT1*, *BCAT2*, and *SLC6A15* in MASLD but not in control participants or those with metabolic diseases, suggesting a MASLD-specific, CMS-sensitive suppression in BCAA uptake and catabolism. Adjustment for obesity markers (adiponectin/leptin ratio) or liver damage (AST) negated the CMS–genetic relationship. CMS-linked disruption of BCAA metabolism was correlated with insulin resistance (HOMOIR), liver damage (AST/ALT), and adipose inflammatory markers (adiponectin/leptin) in participants with MASLD. Adjustment for blood BCAAs, insulin resistance, and obesity abrogated the CMS-based prediction of MASLD risk. Collectively, the data suggest that the multifaceted interplayers, such as obesity and insulin-resistance-associated BCAA metabolism, in part, if not all, mediated CMS-associated MASLD risk.

The molecular mechanisms underlying the association of CMS with abnormal BCAA metabolism and MASLD have not been fully elucidated. Altered plasma BCAAs and nutrient abundance have been well-established as activating mammalian target of rapamycine complex 1 (mTORC1) signaling pathways, which regulate whole-body energy homeostasis [63], and critical amino acid sensors that regulate energy metabolism in the liver [64]. As a central regulator of amino acid metabolism, mTORC1 is closely associated with AMPK and energy metabolism. Akt and PI3K, which are upstream of mTORC1, activate hepatic cell insulin receptors. Through whole-system experiments and model analysis of insulin signal transmission, the attenuation of mTORC1-to-IRS1 feedback is a major mechanism underlying insulin resistance in obesity and diabetes [65]. Network signaling pathways, such as PI3K/AKT/mTOR, AMPK/mTORC1, and mTORC1/SREBP1c/DNL, regulate lipid metabolism, insulin resistance, oxidative stress, and inflammation in MASLD [34,66,67]. This study is the first to characterize the mTORC1/AKT/IRS1/AMPK signaling regulators of CMS-sensitive, MASLD-responsive disruption to BCAA metabolism in patients with MASLD. Consistent with recent evidence, we observed that low choline intake was associated with reduced *mTORC1-IRS-Akt* expression, which coincided with an impaired expression profile of BCAA catabolism, elevated HOMOIR, and obesity-related inflammation. In patients with MASLD and obesity, decreased Pch levels were correlated with reduced *AKT1* and *mTOR* expression, coinciding with impaired *BCAT1*, *BCKDK*, and *SLC6A15* expression and elevated BCAAs in patients with lean MASLD. Given that adjustment for mTORC1 expression and BCAA levels negated the observed Pch-responsive and low choline intake-associated MASLD risk, our findings regarding blood metabolites and genetic coexpression network signatures highlight the critical role of mTOR signaling and BCAA catabolism in CMS-responsive MASLD risk.

Pch, a previously proposed noninvasive biomarker for early-stage MASH, predicted a biphasic responsive curve for MASLD risk in our cohort [68,69]. Numerous unfavorable metabolic phenotype changes related to high Pch in our cohort confirm the findings of other human and rodent studies indicating an association between increased Pch levels and unfavorable cardiovascular risk factors [31], insulin resistance [70], and obesity [35]. Our findings are aligned with a National Health and Nutrition Examination Survey report indicating that high choline intake is associated with increased fibrosis risk in patients with overweight or moderate obesity and MASLD [27]. Conversely, Pch levels below the designated cutoff predicted an increased risk of MASLD in our cohort, which is aligned with a reported association between decreased dietary choline intake and an increased risk of MASH in normal-weight Chinese women [24]. Adjustment of our models for blood BCAA and mTORC1 signaling abrogated the linear and nonlinear association of Pch with MASLD risk. Although the wide 95% CI observed beyond the Pch cutoff challenges the observed risk relationship, a similar biphasic risk association of dietary choline intake with MASLD risk prediction was negated by adjustment for blood BCAA and *mTORC1* signaling. Collectively, our data suggest that blood BCAA and *mTORC1* signaling may mediate the predictive power of high Pch and choline intake for MASLD.

A fourth mechanistic possibility underlying the associations observed in this study is that TMAO, a gut-flora-dependent metabolite of choline, contributes to the adverse events experienced by individuals with unfavorable choline intake and/or Pch levels. Several studies have demonstrated a positive association of TMAO with inflammatory pathways, type 2 diabetes mellitus, cardiovascular diseases, and obesity [71,72,73,74]. Randrianarisoa et al. reported a mechanistic link between TMAO, BMI, insulin resistance, visceral fat mass, and liver fat content [75]. In a large sample of hospital- and community-based Chinese adults, Chen et al. observed that circulating TMAO levels and two of its nutrient precursors (choline and betaine) were positively associated with the presence and severity of MASLD [76]. In Mexican patients with obesity, especially those with type 2 diabetes mellitus, circulating TMAO levels were associated with MASH [77]. In our study, decreased choline intake was correlated with elevated blood TMAO levels (r = −0.45, *p* < 0.05), which in turn was associated with reduced abundance of lymphocytic IRS1 transcripts (r = −0.74) and dysfunctional amino acid metabolism in BCAA and an upregulated urea cycle in control patients. Notably, the association of CMS and TMAO with amino acid metabolic disorders was not observed in patients with MASLD. CMS-sensitive steatosis and dysfunctional BCAA metabolism were not associated with TMAO, suggesting that other contributing factors outweigh choline deficiency in blood TMAO concentrations. Although TMAO is a blood metabolite of dietary choline, gut microbiota can also regulate blood TMAO levels, impairing hepatic ability to synthesize TMAO from TMA and altering the kidney clearance rate [78], all of which may also be mechanistically linked to obesity and insulin resistance pathogenesis [71,72,73,74]. Functional studies of hepatic TMAO metabolism and the threshold intake level of CMS that induces dysbiosis are required to clarify the association of TMAO with steatosis-linked metabolic disorders.

The blood lymphocytic transcriptional profile of CMS-responsive disruption to BCAA catabolism may reflect systemic multiorgan imbalance in the PFAA metabolism of patients with MASLD. It has been reported that skeletal muscle metabolizes BCAAs through highly expressed BCAT2 to harvest energy and transfer nitrogen for glutamate formation. Glutamate then drives pyruvate transamination to alanine in muscle, facilitating ammonia transfer to the liver, where BCAT1 is highly expressed [60]. White et al. used static metabolomics and stable isotope tracing technologies to demonstrate that obesity-related BCAA elevation paired with reduced BCAT1 expression in the liver may activate a glycine-depleting, interorgan pyruvate–alanine shuttle to process the excess ammonia generated through skeletal BCAA catabolism. For patients with obesity-related MASLD, glycine becomes a critical source of pyruvate through the combined actions of serine-hydroxymethyltransferase and serine dehydratase in the liver. The intense use of glycine/serine to support the pyruvate–alanine cycle and the production of glutamate/glutamine to transfer BCAA-derived ammonia from skeletal muscle to the liver may lead to hepatic serine/glycine depletion and elevated blood BCAA levels. We found that the deregulated lymphocytic *BCAT1*/*BCAT2*/*mTORC1* signaling expression profile of patients with obesity-MASLD was significantly associated with CMS markers, blood PFAA (BCAAs, Ser, Gly, Glu, and GSG index) imbalance, blood clinical markers, or/and abnormal body adiposity. Such functional correlation between leukocyte transcripts and CMS/blood PFAA/MASLD risk markers robustly exists in control subjects, metabolic disorders subjects, and subjects with low choline intake (below AI intake). Given that adjustment for the lymphocytic BCAA catabolic and signaling transcripts attenuated the association of CMS with MASLD risk, our data suggest that blood transcriptional markers may, in part, if not all, reflect CMS-sensitive BCAA pathways in hepatic and other multiorgan imbalance pathways involved in MASLD risk development. Whether the lymphocytic transcriptional changes alone equated to altered protein activity or metabolic flux requires further functional validation. The use of lymphocytic transcript profile for surrogates of hepatic and multiorgan pathways in MASLD development warrants studies.

## 4. Method and Materials

### 4.1. Study Design

This is a nested case–control study to include 125 participants from the Methyl Nutrition and Metabolic Disorder-Associated MASLD (MeNuNMAS) cohort [21], who underwent ultrasound screening to receive CMS trait assessment, targeted plasma metabolomics, as well as transcriptomics profiling. The recruiting protocol of the MeNuNMAS cohort has been described elsewhere [21]. In brief, participants were recruited from the Taiwan Ministry of Health and Welfare-Affiliated Taipei Hospital and Fu-Jen Catholic University Hospital, Taiwan. MASLD was diagnosed by the presence of hepatic steatosis (HS) (liver fat > 5%) via imaging, alongside at least one cardiometabolic risk factor (e.g., obesity, hypertension, and diabetes). Initially, we selected 125 subjects with ultrasound-based diagnosis of moderate or severe fatty liver alongside at least one cardiometabolic risk factor (e.g., obesity, hypertension, and diabetes) [1,2,3] as the MASLD group (n = 66) and the age/sex-paired non-MASLD group (n = 59) from the MeNuNMAS cohort. Upon the availability of blood samples for metabolomics, transcriptomics, as well as CMS index measurement, the final included subjects in the present study were 48 MASLD cases and 53 non-MASLD cases for the subsequent statistical analysis of each MASLD risk parameter, as shown in Supplementary Table S1. Study protocols were approved by the Institutional Review Board of Fu-Jen Catholic University (FJUH IRB-C108075, IRB-0019-0021), with written informed consent obtained. Recruitment and biospecimen collection took place from January 2020 to December 2023.

### 4.2. CMS Traits Analysis

Overnight fasting blood was collected from the peripheral venous blood (20 mL) of each subject using EDTA-coated evacuated tubes. To separate blood components, the whole blood was centrifuged at 3000 rpm for 20 min at 4 °C. Following centrifugation, the upper layer of plasma was used for MDN trait analysis as previous described [25]. In brief, plasma folate levels were measured using radioimmunoassay kits (Becton Dickinson, Franklin Lakes, NJ, USA), and plasma homocysteine levels were determined using a commercially available fluorescence polarization immunoassay kit and an Abbott 130 AxSYM system (Becton Dickinson, Franklin Lakes, NJ, USA). Plasma trimethylamine-N-oxide (TMAO) concentrations were quantified using a commercial sandwich ELISA kit (Human TMAO ELISA Kit, Cat. No. ELK8356, ELK Biotechnology, Wuhan, China). Plasma samples were processed according to the manufacturer’s protocol, and absorbance was measured at 450 nm with concentrations calculated from a standard curve [79]. Choline intakes were assessed by use of a validated semi-quantitative food frequency questionnaire (qFFQ) as described in previous studies [25,80]. The plasma betaine-to-choline ratio (Pbcr) was derived from plasma concentrations of betaine and free choline, which were measured using liquid chromatography/electrospray ionization–isotope dilution mass spectrometry, as described previously [81].

### 4.3. Measurements of Body Composition, Body Fat Distribution, and Obesity

Body composition and adiposity parameters were evaluated using a multi-frequency bioelectrical impedance analysis (BIA) system (InBody 270; SELVAS Healthcare, Seoul, Republic of Korea) [25]. To ensure data integrity and measurement precision, participants were positioned barefoot on the base electrodes while maintaining firm contact with the tactile thumb electrodes. The device employs segmental multi-frequency technology to differentiate tissue types based on their specific electrical conductivity, enabling the precise quantification of skeletal muscle mass (SMM) and body fat mass (BFM). By assessing lean and adipose tissue distribution across five anatomical segments (the trunk and four extremities), the system calculated the percentage of body fat (PBF) and total adiposity. All assessments were conducted under strictly standardized conditions—including a mandatory fasting period and supervised electrode placement—to minimize inter-individual variability and ensure the clinical reliability of the musculoskeletal metrics.

#### Genetic Polymorphism, Global Epigenetic Markers, and Transcriptional Analysis

Overnight fasting blood was collected from the peripheral venous blood (20 mL) of each subject using EDTA-coated evacuated tubes. To separate blood components, the whole blood was centrifuged at 3000 rpm for 20 min at 4 °C. Following centrifugation, the buffy coat (leukocytes) was used for DNA extraction (DNeasy Tissue Kit, Qiagen Science, Germantown, MD, USA). Genomic DNA was isolated from the buffy coat through a sequence of enzymatic digestion and chemical precipitation. Briefly, cells were lysed at 60 °C, followed by RNA and protein removal using RNase A and protein precipitation buffer. DNA was then precipitated with isopropanol, washed twice with 70% ethanol, and air-dried at 60 °C. The final DNA pellet was rehydrated in 100 μL of hydration buffer at 60 °C for 45 min.

Genotyping of *PEMT rs7946* [21] and *MTHFR C677T* [82] was performed using real-time PCR (Chromo 4, Bio-Rad, Hercules, CA, USA) with specific primers and TaqMan probes (Applied Biosystems, Foster City, CA, USA) (Supplementary Table S2). The PCR reaction mixture (20 mL total volume) contained 10 mL of KAPA PROBE FAST qPCR Master Mix, 100 ng of genomic DNA, and 0.4 mL of a customized primer–probe premix (comprising 10 mM each of forward primer, reverse primer, and both probes). Thermal cycling was initiated with an enzyme activation step at 95 °C for 3 min, followed by 40 cycles of denaturation at 95 °C for 10 s and annealing/extension at 60 °C for 40 s. Fluorescence signals were captured at the end of each cycle for genotype calling. *LINE-1* DNA (500 ng) underwent bisulfite conversion using the EZ DNA Methylation-Gold Kit (Zymo Research, Irvine, CA, USA). Briefly, DNA was denatured at 98 °C for 10 min and converted at 64 °C for 3.5 h. Following desulphonation and purification via spin-column centrifugation (10,000× *g*), the converted DNA was eluted in sterile water, quantified via High-Resolution Melting (HRM) analysis on a LightCycler system (Roche Diagnostics, Germany) for *LINE-1* methylation levels [83]. RNA extraction and reverse transcription polymerase chain reaction were performed following established protocols [30] (Supplementary Table S3).

### 4.4. Ultra-Performance Liquid Chromatography (UPLC)-Based Amino Acid Measurement

This nested case–control study included MASLD and age- and sex-matched non-MASLD subjects, as the controls, for targeted metabolomics profiling on blood free amino acids composition. The plasma samples were collected and stored at −80 °C until assayed. The plasma samples (100 µL) were precipitated by adding an equal volume (100 µL) of 10% sulfosalicylic acid containing an internal standard (norvaline 200 µM) [84]. After protein precipitation, the samples were vortexed and centrifuged at 12,000× *g* for 10 min at room temperature. After the samples were centrifuged, 20 µL of the supernatant was mixed with 60 µL working buffer (borate buffer, pH 8.8). The derivatization was initiated by adding 20 µL of 10 mM AQC (6-aminoquinoly-N-hydroxysuccinimidyl carbamate) in acetonitrile. After 10 min of incubation, the reactant was mixed with an equal volume of Eluent A (20 mM ammonium formate/0.6% formic acid/1% acetonitrile) and analyzed using the ACQUITY UPLC System. The AQC derivatization reagent was obtained from the Waters Corporation (Milford, MA, USA) [85].

Liquid chromatographic separation was achieved on a ACCQ-TAG ULTRA C18 column (1.7 μm, 2.1 × 100 mm, Waters Corp.; Milford, MA, USA) using an ACQUITY TM Ultra Performance Liquid Chromatography (UPLC) system (Waters Corp.). The column was maintained at 55 °C, and the flow rate was set at 0.6 mL/min. Solvent A was 0.8% formic acid and 20 mM ammonium formate in 1% acetonitrile and solvent B was acetonitrile. Analytes were eluted from the column using with a linear gradient: 0–2.5 min: 0.1% B; 2.5–3.2 min: 0.1–2% B; 3.2–4.1 min: 2–9% B; 4.1–4.9 min: 9–10% B, keep 1 min; 5.9–7 min: 10–20% B; 7–8.24 min: 20–21.2% B; 8.24–8.55 min: 21.2–90% B, keep 0.6 min; and 1 min for re-equilibration. The detection was set at 260 nm using a sampling rate of 20 points/s. Injection volumes for all samples and standards were 2 μL. Detector was performed on a Waters PDA (Waters MS Technologies, Manchester, UK) operated at 260 nm. The concentrations of the 22 amino acids were quantified using a standard curve ranging from 25 µM to 500 µM. The limits of detection (LOD) and limits of quantification (LOQ) for these 22 amino acids are listed in a table. The targeted metabolome-wide accurate quantification was presented in Supplementary Table S4.

### 4.5. Weight Gene Coexpression Network Analysis

To explore the systemic correlation patterns among metabolites, PFAAs exposure raw data were processed using the WGCNA package (version 1.74) in R [86], as previously applied in metabolomics studies. A soft-threshold power of 5 was selected using the pickSoft Threshold function to meet scale-free topology criteria. An adjacency Pearson correlation matrix was constructed and converted into a Topological Overlap Matrix (TOM) to measure the network interconnectedness and minimize the effects of noise and spurious correlations. Hierarchical clustering was performed based on TOM-based dissimilarity. Coexpression modules were identified by hierarchical clustering and dynamic tree cutting (deepSplit = 2, min, ClusterSize = 2, and cutHeight = 0.99), resulting in three distinct modules. TOM-based dissimilarity was used to generate a dendrogram and heatmap of network structure [87]. The biological significance of hierarchical clustering of module eigengenes (mergedMEs) was used to assess associations between PFAAs and MDN clinical traits, identifying MASLD-related modules for further pathway analysis. Hub metabolites were identified using CytoHubba (Cytoscape, version 3.10) and the maximal clique centrality method [88].

### 4.6. Statistical Analysis

Mann–Whitney *U*-test was performed for non-normal continuous data, which were presented as median (25–75 percentile). And the χ2 test or Fisher’s exact test was performed for categorical variables. Significance was set at *p*-values < 0.05. The statistical analyses were performed using SPSS V.20.0 for Windows (SPSS, Chicago, IL, USA). Univariate correlations were determined using the Spearman rank correlation test. For multiple regression analyses, adjustments were made for covariates, which were normalized through log transformation. Restricted cubic spline regression with four knots (0.10, 0.35, 0.65, and 0.90) was performed to investigate the nonlinear association between HS risk and plasma choline metabolites index; this analysis was conducted using R (version 4.2.1) [89,90]. ROC curve plotting was used to assess the disease diagnostic ability of the model [91]. Correlation networks were mapped using MetScape version 3.1.3 [92]. Restricted cubic splines were employed to investigate the plausible nonlinear associations between dietary choline intake and its related metabolites and the risk of NAFLD. Moreover, a receiver operating characteristic curve analysis was conducted, facilitating the calculation of AUC to assess the discriminative capacity of PFAA metabolites in predicting the occurrence of MASLD.

### 4.7. Metabolomics Statistical Analysis

A correlation network was created to visualize the relationships between gene expression and metabolites. Partial correlations were calculated using the debiased sparse partial correlation algorithm to analyze associations related to PfCH responsiveness, NAFLD sensitivity, and disease subtypes (OB and/or insulin resistance). Networks were mapped with MetScape v3.1.3 [92]. Pearson correlation was used to evaluate relationships between leukocyte transcript levels and plasma metabolites. A heatmap was constructed based on correlation results and generated using GraphPad Prism 9.

## 5. Conclusions

Our findings highlight the strong effect of CMS, including low choline intake and Pbcr, on the genetic–plasma metabolomic network, particularly in the context of systemic metabolic disruption affecting BCAAs, Ser/Gly metabolism, and the transcriptional network signatures of BCAA catabolism and the mTOR signaling pathway. We also identified CMS of altered Pch as a key determinant of obesity-associated BCAA and Ser/Gly signatures, which predicted a biphasic risk curve for MASLD involving the transcriptional network signatures of BCAA catabolism and the mTOR signaling pathway. These insights provide a foundation for follow-up validation studies that can guide the development of targeted nutritional interventions to manage metabolic risk factors for MASLD with obesity.

### 5.1. Study Strengths

We elucidated mediators of the predictive power of choline metabolites for MASLD risk through an integrative approach involving comprehensive choline nutrition assessment, targeted blood metabolomic profiling, and genetic polymorphism and transcriptional expression analysis. These strategies allowed us to identify key blood biomarkers and establish novel mechanistic connections between dietary intake, nutrigenomics, and nutrigenetic processes in human MASLD risk prediction. Additionally, the plasma metabolite panel includes TMAO, a byproduct of microbiota digestion of dietary phosphatidylcholine that has been linked to metabolic disorders. Further characterization of TMAO can deepen understanding of choline metabolism and clarify the divergent effects of Pbcr on PFAA signatures associated with obesity and insulin resistance in control individuals and those with MASLD. The insights gleaned through this integrative approach are not achievable through the conventional evaluation of single components, offering a novel platform for prevention and intervention in precision nutrition medicine. Although our discovery cohort was small, our systematic approach enabled comprehensive metabolomic screening, through which we identified a robust set of PFAA modules associated with CMS traits, metabolic risk factors, and transcriptional mediators.

### 5.2. Study Limitations

This nested case–control study is subject to certain limitations, including selection bias and small sample size. The small sample size relative to model complexity is the top limitation. In this case, overfitting with background noise and random fluctuations may occur to impact poor MASLD prediction by the comprehensive CMS index. Without adequate cross-validation, prediction models are highly susceptible to these problems, which could translate to poor real-world applications. The present study suffers from the potential overfitting and lack of multiple-testing correction, which warrants human RCT intervention studies for adequate cross-validation. The limited lymphocytic specimen of the study participants did not allow us to perform Western or/enzymatic assay to validate translational functionality. Transcriptional changes alone do not equate to altered protein activity or metabolic flux. Functional validation on transcriptional regulators and signaling molecules in CMS-related BCAA catabolism warrants studies. The use of leukocyte transcripts as surrogates for hepatic pathways further weakens causal mechanistic inferences. The cross-sectional design and use of a specific patient population limited our ability to draw causal inferences or generalize our results. A longitudinal, cost-effective factorial design with independent discovery and replication cohorts is warranted to validate major CMS-responsive and obesity-sensitive metabolite markers for MASLD management.

## Figures and Tables

**Table 1:** Pbcr and obesity-linked MASLD risk involved blood genetics and metabolite coexpression network signatures ^1–4^.

	WHR_OB BCR < 4.27	WHR_NOB BCR < 4.27	WHR_OB BCR > 4.27	WHR_NOB BCR > 4.27
MAFLD/Control	23/7	10/15	7/7	5/23
Model 0	15.114 (4.182–54.631) *	3.067 (0.874–10.760)	4.600 (1.105–19.141) *	1
Model 1	13.660 (3.651–51.105) **	2.635 (0.729–9.530)	4.783 (1.073–21.315) *	1
Model 2	18.664 (4.359–79.911) **	2.586 (0.668–10.012)	5.390 (1.043–27.853) *	1
Model 3	5.656 (1.052–30.401) *	1.075 (0.220–5.239)	3.980 (0.610–25.945)	1
Model 4	1.156 (0.142–9.424)	0.813 (0.131–5.050)	1.092 (0.096–12.419)	1

^1^ Data was presented with OR (95% CI); Model 0: crude model. ^2^ Adjustment Models: Model 1: adjusted for age and gender; Model 2: M1 + dietary choline intake, *BCAT1*, *BCKDK*, and *mTORC1*; Model 3: M2 + TG; and Model 4: M3 + HOMA-IR, * *p* value < 0.05, ** *p* value < 0.01. ^3^ Obesity criteria is a waist-to-hip ratio (WHR) > 0.9 for men and > 0.85 for women. ^4^ Plasma betaine-choline ratio (BCR) cutout (4.27) was set at ROC prediction levels of the study subjects.

## Data Availability

The raw data supporting the conclusions of this article will be made available by the authors on request.

## Supplementary Materials

The following supporting information can be downloaded at: https://www.mdpi.com/article/10.3390/ijms27104186/s1.
