# Supplementary figures and images for: Blood Focused-Metabolomics and Transcriptomics Uncover Non-Linear Risk Association of Inadequate Dietary Choline Intake-Linked Metabolic Stress with MASLD Through Amino Acid Biomarkers, BCAA and MTORC 1/AKT1/IRS1 Mechanistic Mediators: A Nested Case–Control Study
Source: Int J Mol Sci. 2026 May 8;27(10):4186. doi: 10.3390/ijms27104186 (PMC13206527; doi:10.3390/ijms27104186)

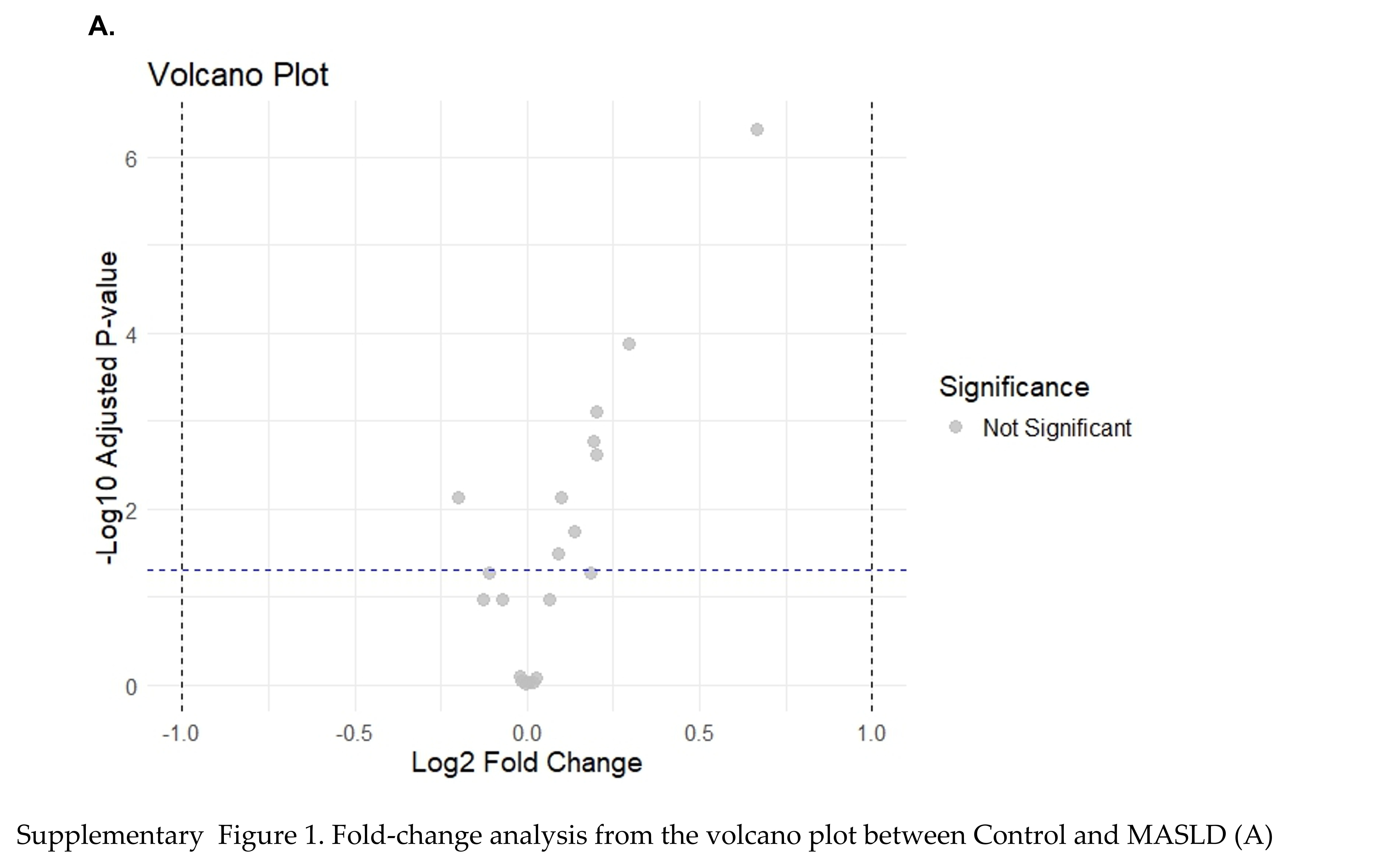

Supplement: Supplementary file 1 [file ijms-27-04186-s001.zip › Fig1 S.jpg]

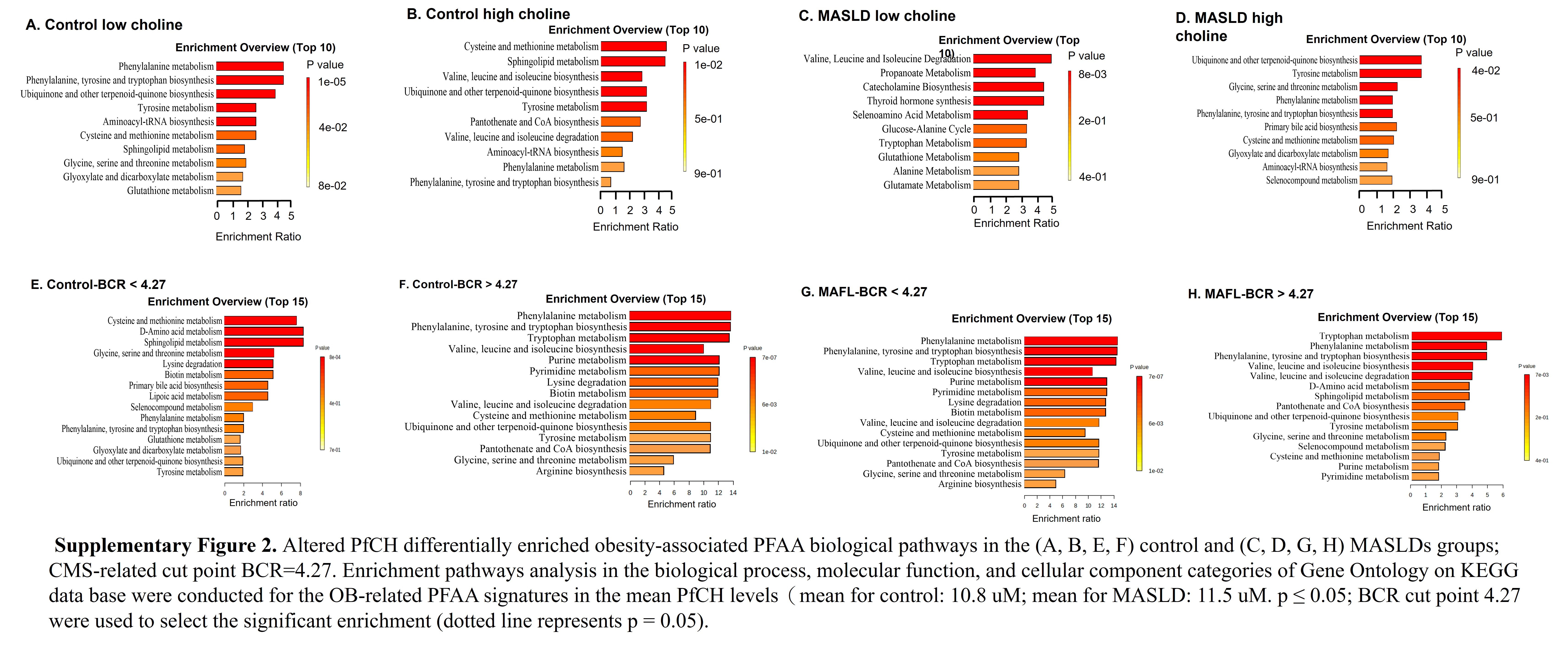

Supplement: Supplementary file 1 [file ijms-27-04186-s001.zip › Fig2 S.jpg]

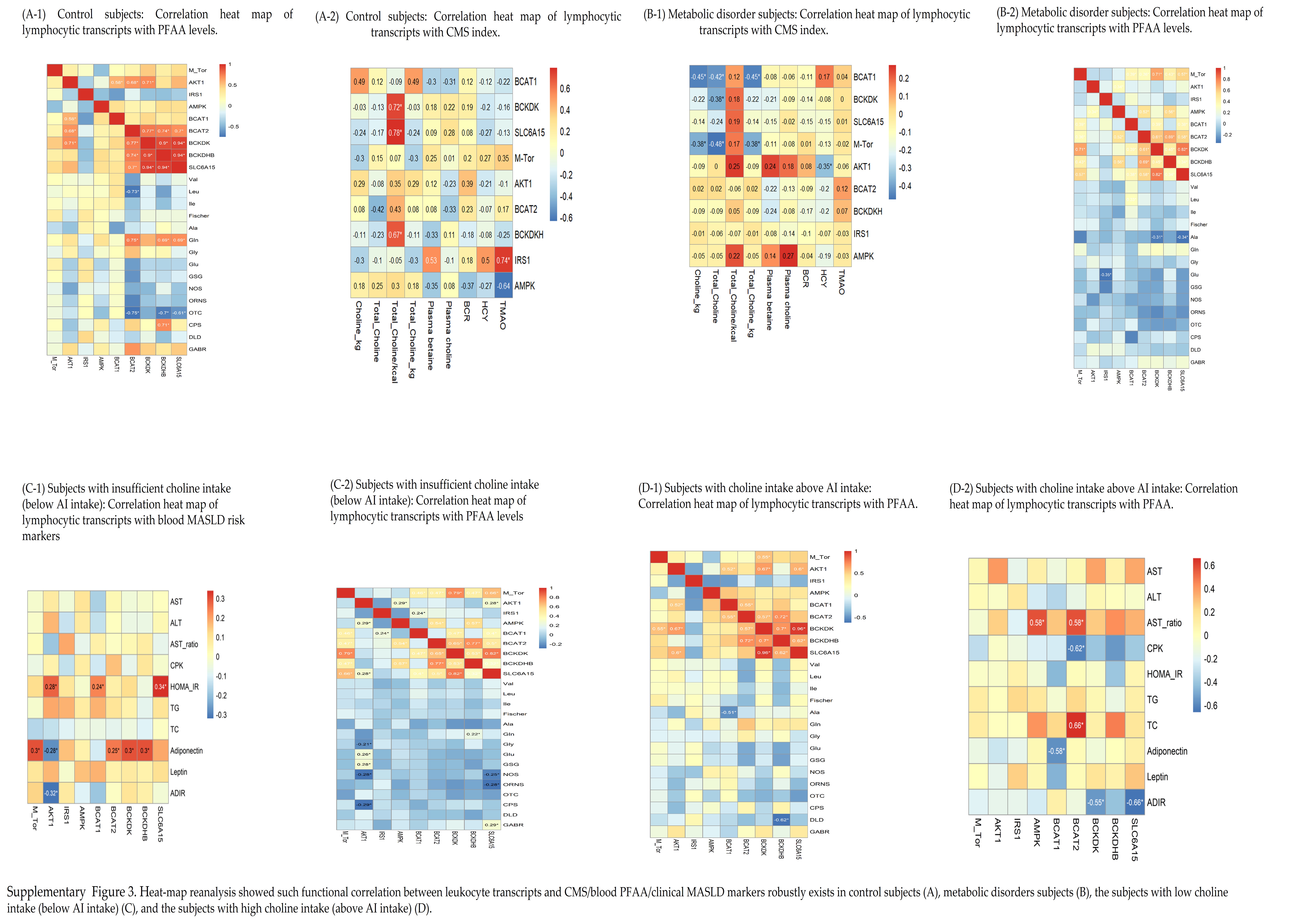

Supplement: Supplementary file 1 [file ijms-27-04186-s001.zip › Fig3 S.jpg]

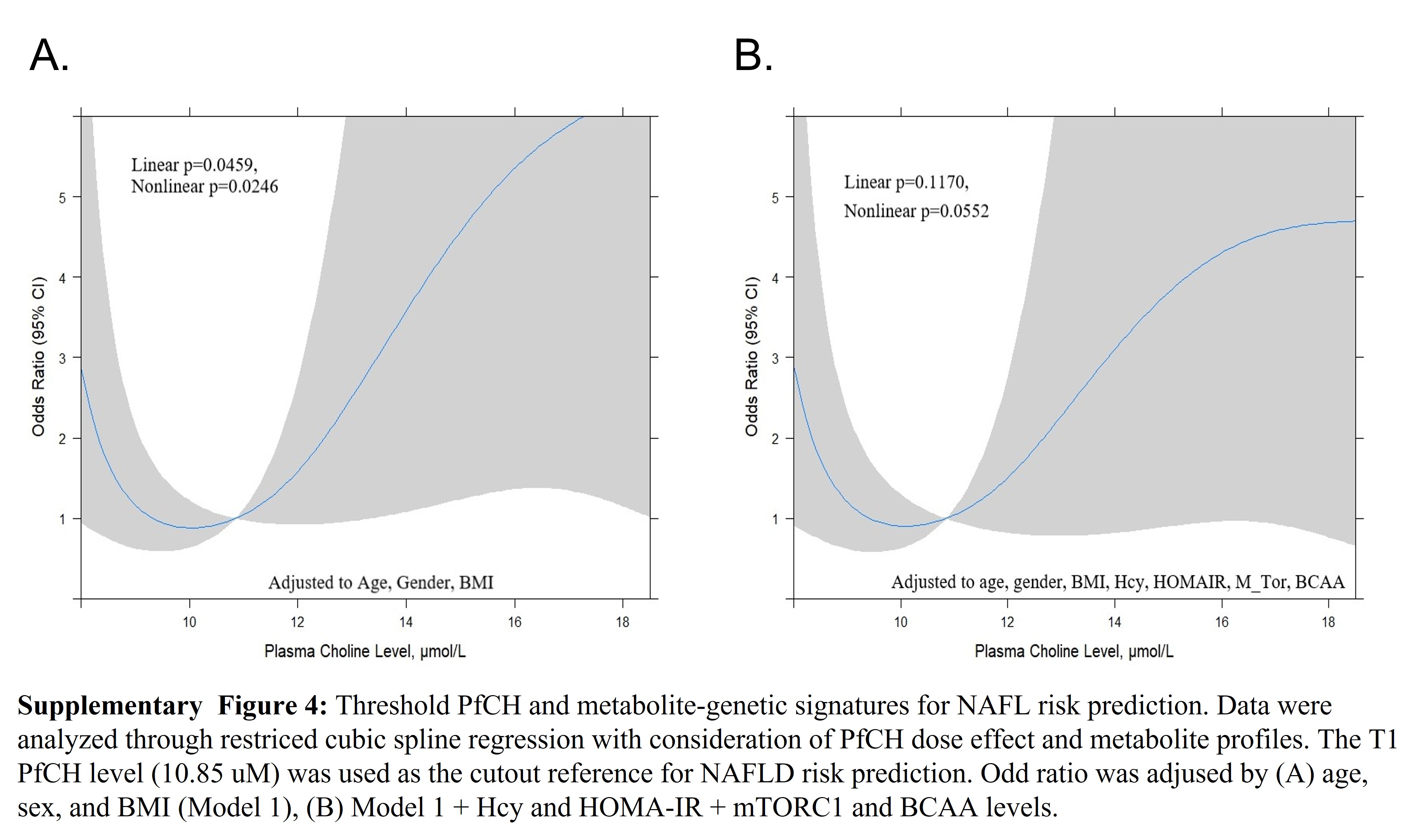

Supplement: Supplementary file 1 [file ijms-27-04186-s001.zip › Fig4 S.jpg]

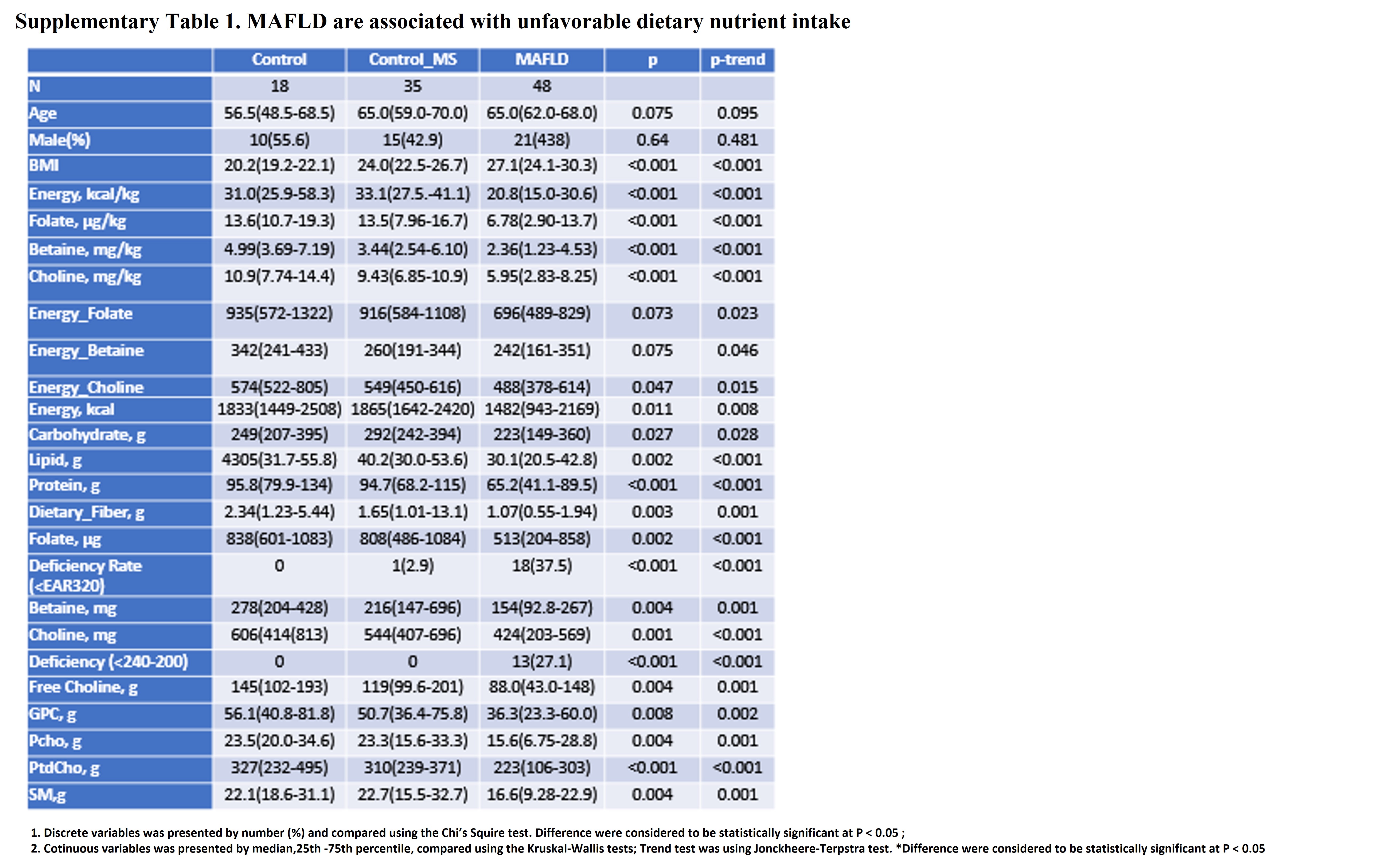

Supplement: Supplementary file 1 [file ijms-27-04186-s001.zip › S tab1.jpg]

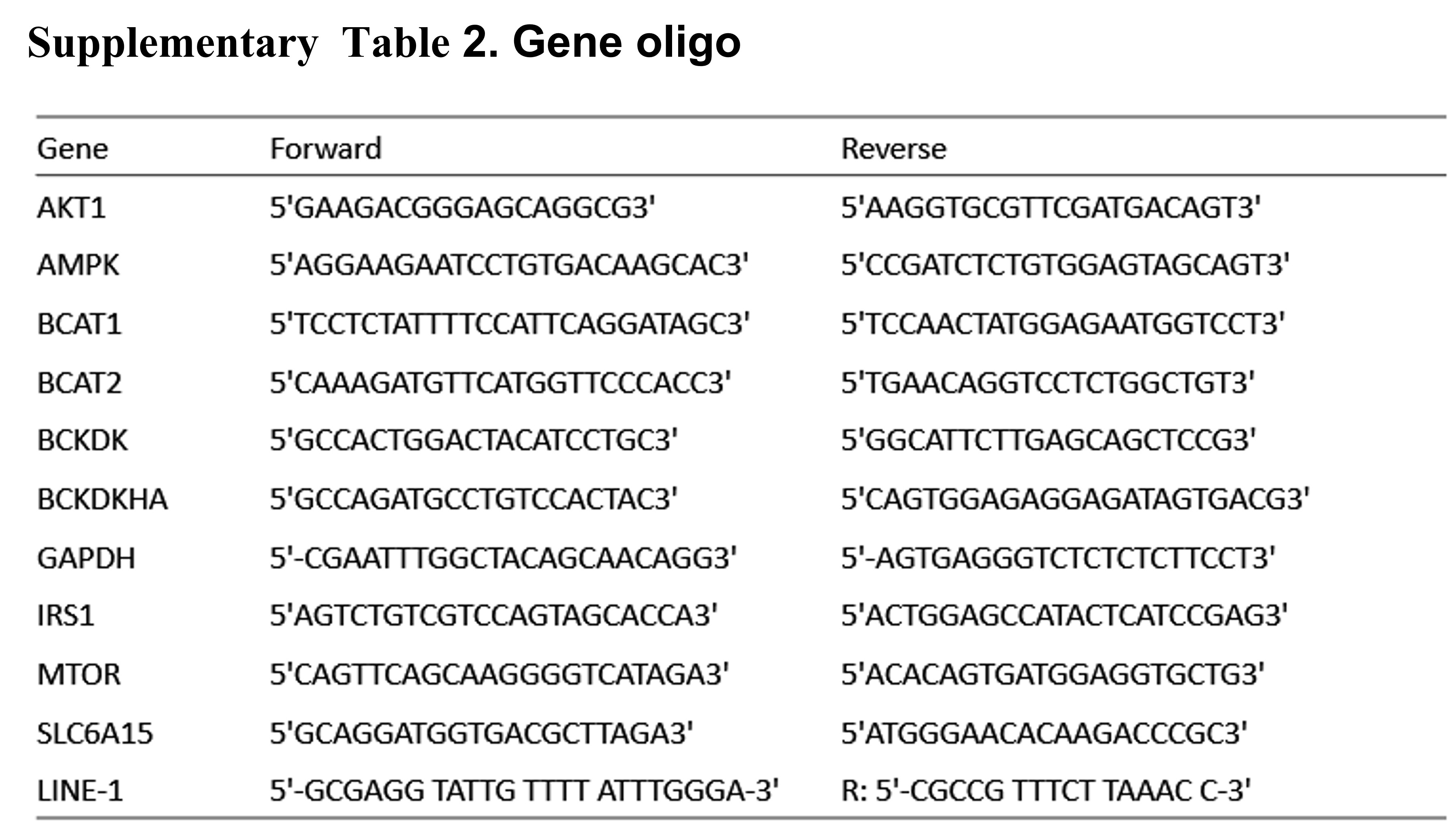

Supplement: Supplementary file 1 [file ijms-27-04186-s001.zip › S tab2.jpg]

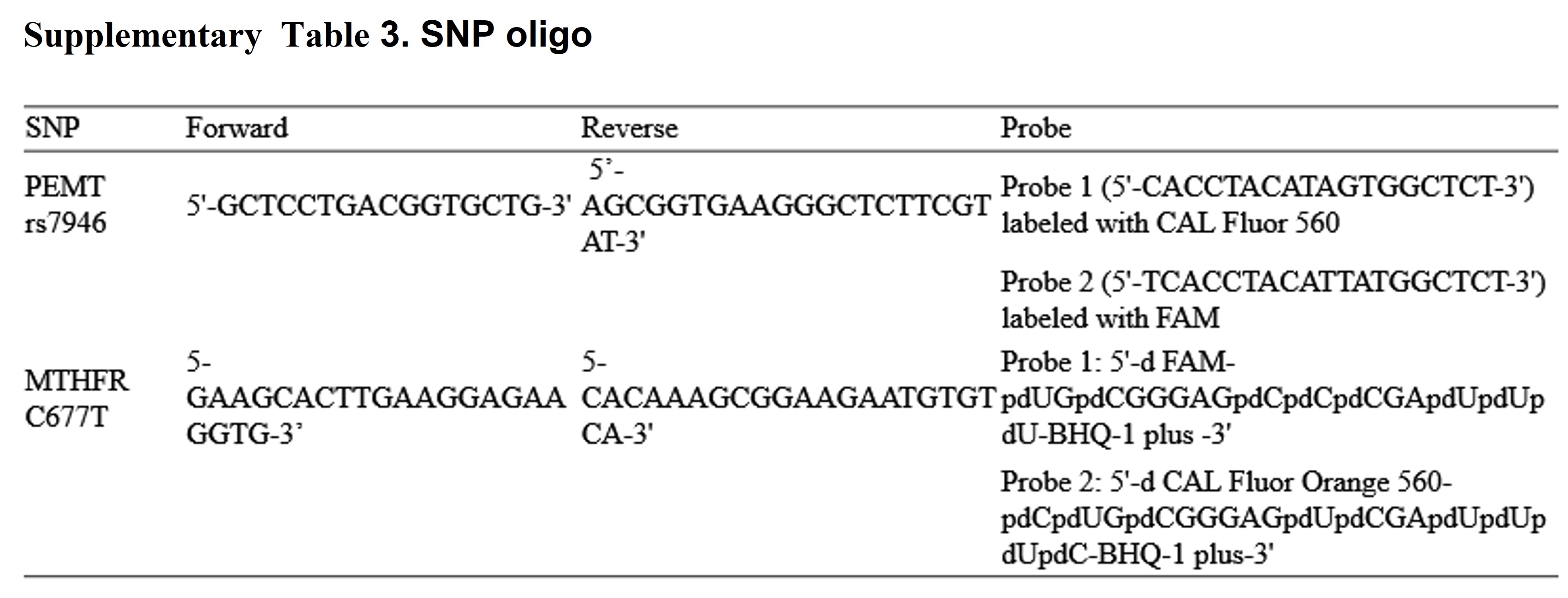

Supplement: Supplementary file 1 [file ijms-27-04186-s001.zip › S tab3.jpg]

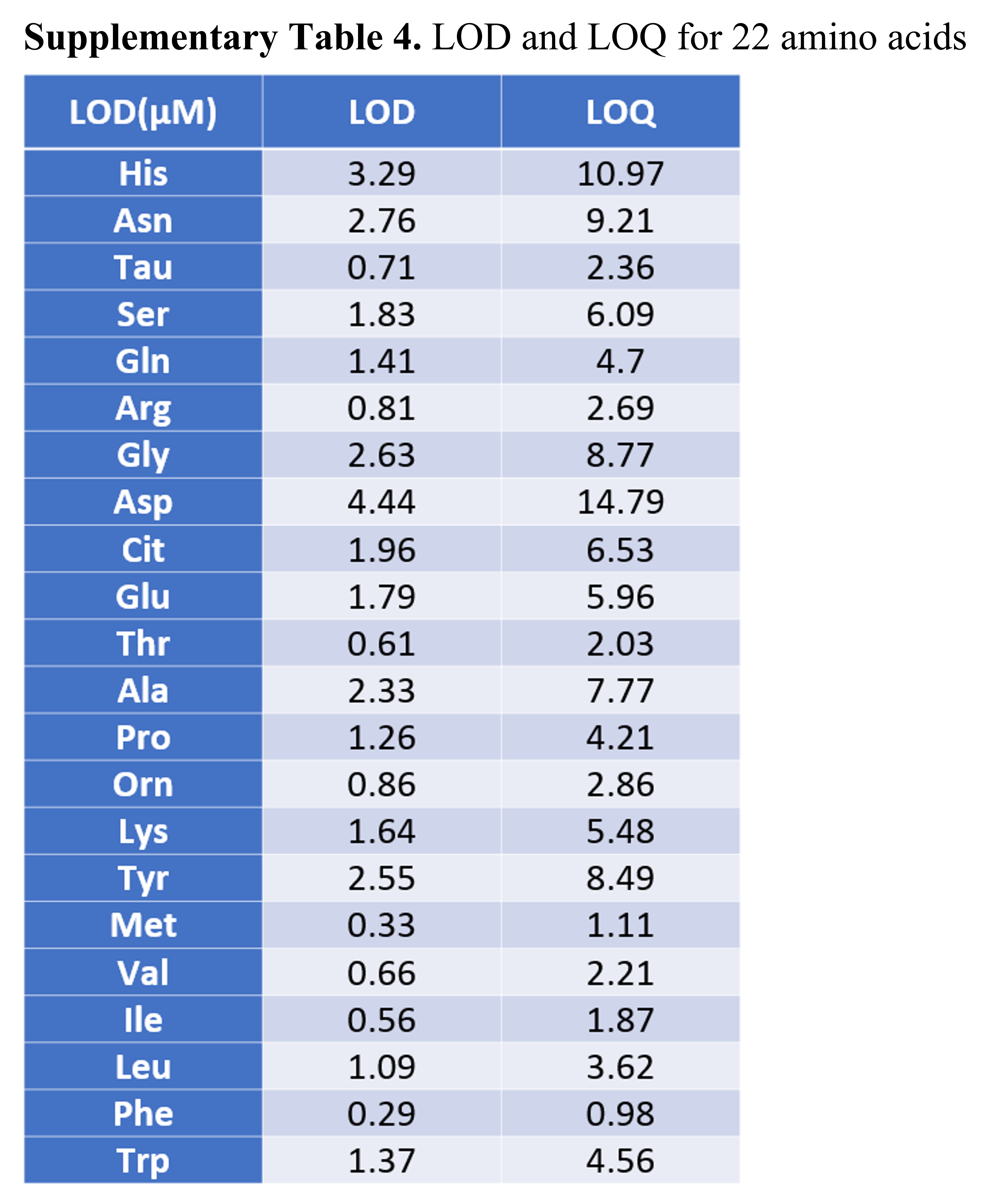

Supplement: Supplementary file 1 [file ijms-27-04186-s001.zip › S tab4.jpg]
